# Microbiome within Primary Tumor Tissue from Renal Cell Carcinoma May Be Associated with PD-L1 Expression of the Venous Tumor Thrombus

**DOI:** 10.1155/2020/9068068

**Published:** 2020-02-18

**Authors:** Michael A. Liss, Yidong Chen, Ronald Rodriguez, Deepak Pruthi, Teresa Johnson-Pais, Hanzhang Wang, Ahmed Mansour, James R. White, Dharam Kaushik

**Affiliations:** ^1^Department of Urology, University of Texas Health San Antonio, San Antonio, TX, USA; ^2^Greehey Children's Cancer Research Institute, University of Texas Health San Antonio, San Antonio, TX, USA; ^3^Department of Cell and Molecular Biology, University of Texas Health San Antonio, San Antonio, TX, USA; ^4^Resphera Biosciences, Baltimore, MD, USA

## Abstract

**Objective:**

To perform a proof of concept microbiome evaluation and PD-L1 expression profiling in clear-cell renal cell carcinoma (cc-RCC) with associated tumor thrombus (TT).

**Methods:**

After IRB approval, six patients underwent radical nephrectomy (RN) with venous tumor thrombectomy (VTT). We collected fresh tissue specimens from normal adjacent, tumor, and thrombus tissues. We utilized RNA sequencing to obtain PD-L1 expression profiles and perform microbiome analysis. Statistical assessment was performed using Student's *t*-test, chi-square, and spearman rank correlations using SPSS v25.

**Results:**

We noted the tumor thrombus to be mostly devoid of diverse microbiota. A large proportion of *Staphylococcus epidermidus* was detected and unknown if this is a surgical or postsurgical contaminant; however, it was noted more in the thrombus than other tissues. Microbiome diversity profiles were most abundant in the primary tumor compared to the thrombus or normal adjacent tissue. Differential expression of PD-L1 was examined in the tumor thrombus to the normal background tissue and noted three of the six subjects had a threshold above 2-fold. These three similar subjects had foreign microbiota that are typical residents of the oral microbiome.

**Conclusion:**

Renal tumors have more diverse microbiomes than normal adjacent tissue. Identification of resident oral microbiome profiles in clear-cell renal cancer with tumor thrombus provides a potential biomarker for thrombus response to PD-L1 inhibition.

## 1. Introduction

The United States anticipates more than 62,000 new renal cell carcinoma (RCC) to be diagnosed each year [1]. RCC can develop intravascular venous invasion commonly referred to as a tumor thrombus, projecting into the inferior vena cava in approximately 4–10% of renal cancer cases [2]. Unfortunately, the five-year overall survival can range from 32 to 69% depending on the presence or absence of metastasis [3–5]. If renal thrombus tumors are left untreated, nearly 87% of these patients will die of renal cancer within a median of 5 months [6]. The tumor thrombus level may not directly affect disease-specific survival; however, the anatomic level of the thrombus can significantly impact surgical complexity [7]. Therefore, new therapy targeting tumor thrombus reduction is needed.

Reports indicate that neoadjuvant chemotherapy with tyrosine kinase inhibitors (TKIs) does not reduce tumor thrombus to improve surgical morbidity [8, 9]. Immunotherapy is quickly being incorporated into advanced kidney cancer protocols with several trials underway [10]. The concept of precision medicine is to target individual tumors with specific therapy, yet requires tumor tissue and knowledge of a particular target [11]. For instance, PD-L1 expression profiling may predict the response of anti-PD-L1 therapy [12], and we have demonstrated that the primary tumor and tumor thrombus have differing PD-L1 expression and that a biopsy of the primary tumor in the kidney is unlikely to predict the PD-L1 expression profile of the tumor thrombus [13]. We hypothesize that the immune function is within the tumor microenvironment. Based on the types of bacteria living within tumors, they may promote intravascular growth of kidney cancer via assisting with immune protection of cancer.

Additionally, bacteria make a variety of compounds that may affect the microenvironment effecting epigenetic signaling. Several groups have discovered that the intestinal microbiome has developed cross-talk with PD-1 and PD-L1 profiling [14]. In this proof of consent study, we investigate the association of various microbiome profiles within the renal tumor tissue associated with specific PD-L1 expression profiles of the tumor thrombus to determine not only the mechanisms to which tumors develop intravascular extension but also potential biomarkers to inform therapy.

## 2. Methods

### 2.1. Population

Six patients were identified with tumor thrombus and consented prior to nephrectomy and thrombectomy. No patient received neoadjuvant chemotherapy. We collected tissue prospectively by flash frozen processing for preservation using standard protocols. The tissue included normal adjacent renal parenchyma, tumor, and thrombus. Additionally, our pathologists performed standard processing as per standard of care. We obtained and recorded data that included demographic, surgical, and clinical outcomes.

### 2.2. RNA-Seq

We performed sequencing with an Illumina HiSeq 3000 system using 100 bp paired-end protocol following the manufacturer's protocol to attain mRNAs of all samples. After we obtained short sequence reads, we aligned them to the UCSC human genome build hg19 using TopHat2 [15]. The bam files from alignment were processed using HTSeq-count to compute the counts per gene in all samples [16].

### 2.3. Bioinformatics and Statistical Analysis

Raw paired-end RNA-seq reads were first filtered for quality (target error rate < 0.25%), Illumina adaptor sequences, and minimum length (95 bp) using Trimmomatic. Bowtie2 searches of the NCBI RefSeq database were performed including fungal, eukaryotic, bacterial, archaeal, and viral members [17, 18]. Pathoscope was extended to include total genome coverage estimates for taxonomic assignment [19, 20]. After assessment of total genome-specific coverage by mapped reads, those microbial members with less than 0.1% average genome coverage were removed from consideration. Additionally, assignments made to the PhiX-174 control genome and *Cutibacterium acnes* were determined to be representatives of contamination and were removed prior to downstream statistical analysis. The paired *t*-test and paired Mann–Whitney *U* test were employed to evaluate statistical significance of differences in taxonomic percentage abundance between groups of interest. The Programmed death-ligand 1 (PD-L1) expression profile cutoff was a two-fold change over adjacent normal kidney tissue. We utilized Student's *t*-test for continuous variables and Fisher's exact test for categorical variables.

## 3. Results

### 3.1. Demographics

Six patients presented with tumor thrombus and underwent nephrectomy with tumor thrombectomy. We display patient demographics in Table 1. All tumors with pT3 and varying levels of tumor thrombus are present. The majority of patients in this small population were of Hispanic ancestry. There were no intraoperative or postoperative deaths. We also show the corresponding PD-L1 expression of the tumor thrombus compared to normal adjacent background expression along with corresponding presence of oral microbiota within the primary renal tumor.

### 3.2. Microbiome Analysis

**Figure 1:**
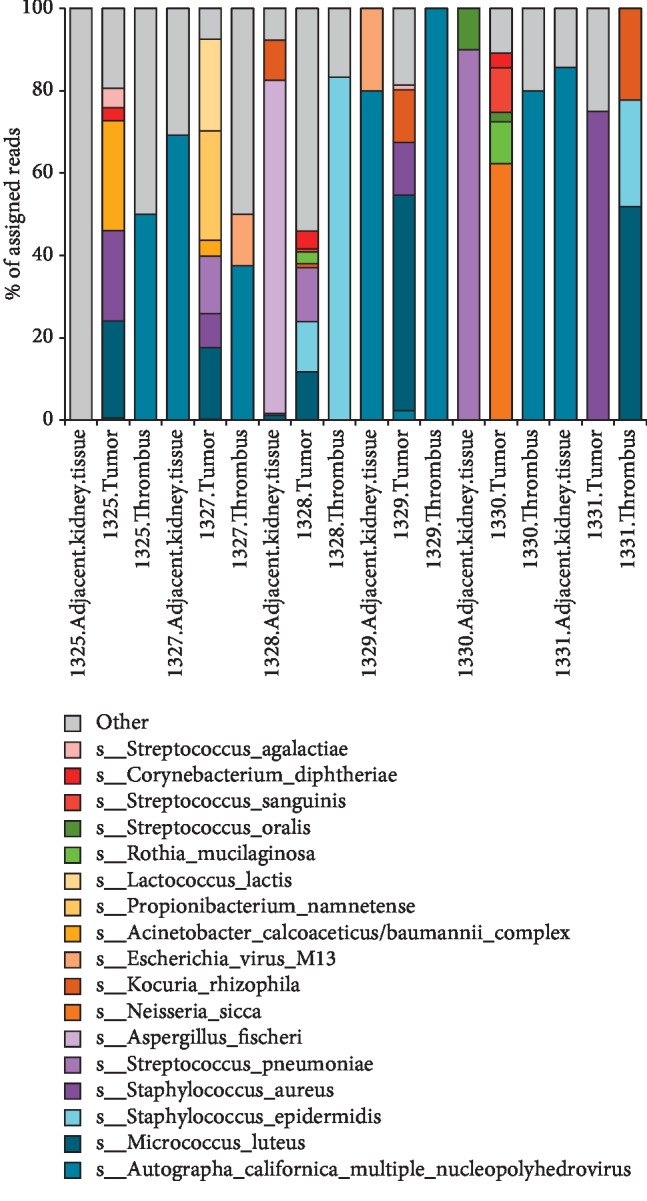
Pathoscope results. Waterfall plot displaying the Pathoscope results identifying the most common microbial genes present in each sample after the removal of PhiX and C. acnes as contaminants.

We analyzed a total of 18 samples from 6 patients. Within our taxonomic profiling, 53.5% of reads were mapped to a reference microbial genome. We removed *PhiX* and *Cutibacterium acnes* as known dominant contaminants prior to analysis. Overview of microbial members detected in each sample is shown as a waterfall plot in Figure 1. We excluded *Cutibacterium acnes* because it was highly represented in each sample and we could then display more clearly the other species identified in the tissues.

**Figure 2:**
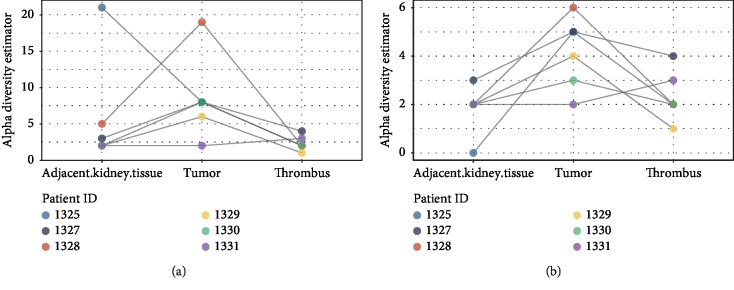
Alpha diversity analysis between locations. (a) shows alpha diversity with an abundance of >1% using an alpha diversity estimator as the comparator. In (b), we display the alpha diversity with an abundance of >5%.

**Figure 3:**
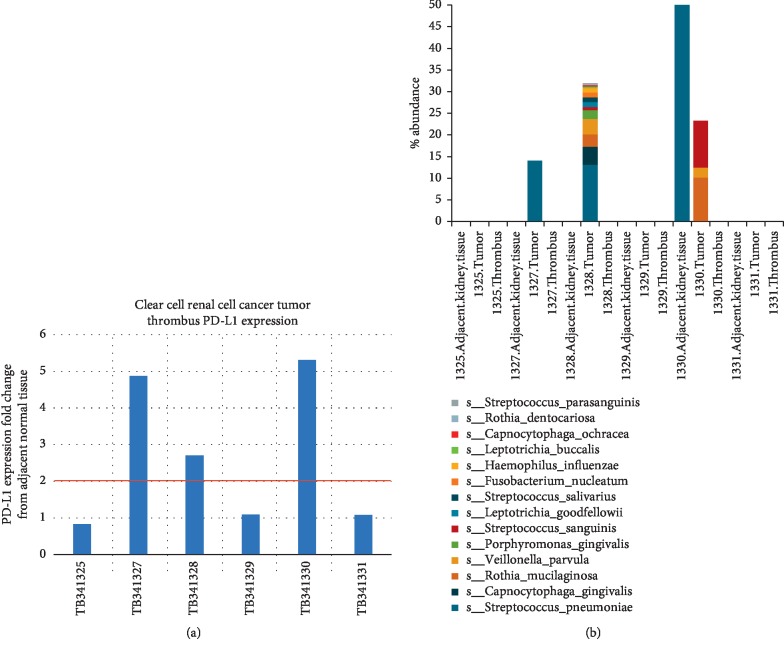
Thrombus PD-L1 expression profile in relation to the presence of oral microbiome within the primary tumor. The top bar graph represents the PD-L1 expression within a renal tumor thrombus compared to background normal adjacent tissue. A two-fold difference is demarcated by the red line. The lower bar graph represents the same tumor specimen's oral microbiome abundance.

Alpha diversity findings (Figure 2) note statistically significant differences between groups over the three locations (normal, adjacent tumor and thrombus; *P*=0.05). We analyzed the findings of both >1% bacterial abundance and >5% bacterial abundance, both noting the tumor with the highest diversity between samples. We note that microbial diversity within tumors is the greatest. We specifically identified *Micrococcus luteus*, *Fusobacterium nucleatum*, *Streptococcus agalactieae*, and *Corynebacterium diphtheriae* to be more abundant within the tumor specimens, as compared to normal adjacent kidney and tumor thrombus. Specifically noting the microbes traditionally assigned to the oral microbiome, oral microbial members of interest are displayed in Figure 3. We noted a single tumor sample with high *Fusobacterium nucleatum* (patient 1330), who also had the highest PD-L1 expression within the tumor thrombus. We identified three subjects with oral microbiome aggregates located within the tumor which were noted to have high PD-L1 expression within the tumor thrombus.

### 3.3. PD-L1 Expression

Examining tumor compared to adjacent normal using a two-fold change or higher cutoff from the normal adjacent PD-L1 expression, we noted three tumors (ID# 0,7, and 8) were of high PD-L1 expression in the tumor thrombus noting 5.3-, 4.9-, and 2.7-fold differences, respectively (Figure 3). We compared the fold-change differences in those with or without the presence of the oral microbiome composite and noted a statistical difference regarding PD-L1 expression specifically in the thrombus (*T*-test, *P*=0.015). Overall, all 3 of those subjects with oral microbiome aggregates located within the tumor were noted to have high PD-L1 expression within the tumor thrombus (Fisher's exact, one-tailed *t*-test; *P*=0.05).

## 4. Discussion

We have discovered a low detectable rate of microbiome profiles within tumor thrombus and higher diversity of microbes within kidney cancer tissue. We have identified that incongruous oral microbiota within primary renal cancers have an association with PD-L1 expression in the propagated intravascular component of renal cancer. If corroborated, this finding could not only serve as a biomarker for PD-L1 in RCC tumor thrombus patients, but may provide insight regarding tumor thrombus formation and the microenvironment. Our small sample size does not allow for extensive analysis or causal inference, but does provide a new potential biomarker to explore in a larger population. The intravascular portion of the renal tumor is not typically biopsied and would be invasive, which is why we focused on the intratumor microbiota profile. Primary renal tumor tissue can be biopsied, but does suffer from genetic heterogeneity and is one of the controversies regarding renal biopsy accuracy for precision medicine and stratification in clinical trials [21–23].

Gut microbiome has been shown to influence tyrosine kinase therapy in renal cancer [24]. However, there is also an evolving landscape of immune checkpoint inhibitors currently undergoing clinical trials in kidney cancer. Several studies in other cancers have reported response rates could be altered by gut microbiome interactions [14, 25–27]. We did not study the gut microbiome; however, previous investigations have shown that intestinal microbiome may have influence on immune therapy. Our investigation focuses on microbiota identified within the tumor. Given the presence of gastrointestinal tract bacteria within a tumor microenvironment, we hypothesize there may be an immunologic cross-talk occurring within that environment. We do not assume that bacteria cause kidney cancer, but it is interesting that migration of nonresident bacteria can accumulate in the tumor microenvironment. The interaction of these bacteria with the immune system may provide insights into how cancer and or microbiota can inhibit immune cell function to create an environment conducive to intravascular propagation of renal cancer.

The identification of resident oral microbiota within tumors is not unique to kidney cancer. Oral pathogens considered in aggregates such as *Fusobacterium nucleatum*, *Parvimonas micra*, and *Peptostreptococcus stomatis* are highly enriched in colorectal cancer [28]. In particular, *F. nucleatum* induced CCL20 protein expression in in vitro colorectal cancer cells and stimulated macrophage activation and migration [29]. *Fusobacterium* was also found in the urine of patients with bladder cancer and considered as a possible protumorigenic pathogen [30]. The authors confirmed 26% (*n* = 11/42) bladder cancer tissues had *Fusobacterium nucleatum* within the tumor. In our sample, we identified two patients with Fusobacterium and both had more than 2-fold expression of PD-L1 within their tumor thrombus. We hypothesize that oral resident microbes have enhanced ability to live in harsh environments and interact with the host immune system better than other organisms. This communication may enhance immune privilege in certain tumor thrombus patients.

Limitations of the current study include the low sample size to produce conclusive evidence of PD-L1 expression of the tumor thrombus and incongruous oral microbiota found in the renal tumor. Our tissue samples were taken directly from tumor removed at the time of surgery and flash frozen. Therefore, in order to expand our hypothesis as a biomarker, we will need to corroborate these finding from formalin-fixed paraffin-embedded (FFPE) samples. Our preliminary data will need corroboration with PDL-1 staining in a larger sample size. In order to secure pathologists and materials, we seek to publish our primary findings and obtain funding for a larger samples size that would include PD-L1 immunohistochemistry and RNA-seq analysis. We present a proof of concept study to guide future microbial tissue studies. Ideally, to confirm PD-L1 response rates, biopsies obtained before PD-L1 randomized clinical trials could provide preliminary data regarding the effect of our findings.

## 5. Conclusion

We have described an association between the presence of the oral microbiome aggregate within primary renal cancer tumors and a potential interaction with the tumor microenvironment. We have suggested that there may be interactions with PD-L1 expression profiles within RCC tumor thrombus that will need further investigation.

## Figures and Tables

**Table 1:** Demographics.

ID^*∗*^	Age	Ethnicity	Laterality	BMI	Tumor size (cm)	Stage	Mayo thrombus level	Fuhrman grade	Thrombus PD-L1 expression^*∗∗*^	Oral microbiome present
5	46	Hispanic	Right	29.1	10.5	T3b, N0, M1	L1	3	0.833	No
7	55	Hispanic	Left	36.7	4	T3a, N0, M0	L1	2	4.874	Yes
8	39	Hispanic	Right	21.6	6.5	T4, N0, M1	L2	4	2.705	Yes
9	69	White	Left	35.6	11	T3a, N0, M0	L1	2	1.092	No
0	46	Hispanic	Left		15	T3b, N0, M1	L2	4	5.304	Yes
1	85	White	Left	26.3	8	T3c, N0, M0	L4	3	1.083	No

^*∗*^ID is the last digit from patient ID (e.g, TB31325). ^*∗∗*^Thrombus PD-L1 expression is a fold-change compared to normal adjacent tissue.

## Data Availability

The data used to support the findings of this study are available from the corresponding author upon request.
